# A brief history of otorhinolaryngolgy: otology, laryngology and rhinology

**DOI:** 10.1016/S1808-8694(15)30132-4

**Published:** 2015-10-19

**Authors:** João Flávio Nogueira Júnior, Diego Rodrigo Hermann, Ronaldo dos ReisAmérico, Iulo Sérgio Barauna Filho, Aldo Eden Cassol Stamm, Shirley Shizuo Nagata Pignatari

**Affiliations:** a MD. ENT resident - Hospital Prof. Edmundo Vasconcelos; b MD. ENT resident - Hospital Prof. Edmundo Vasconcelos; c MD. ENT resident - Hospital Prof. Edmundo Vasconcelos; d Otorhinolaryngologist, Otology Fellow - HCFMUSP, PhD Student in Otorhinolaryngology - HCFMUSP; e PhD in Otorhinolaryngology - UNIFESP, Head of the Otorhinolaryngology Center - São Paulo - Hospital Prof. Edmundo Vasconcelos; f PhD in Otorhinolaryngology - Escola Paulista de Medicina - UNIFESP, Head of the Pediatric Otorhinolaryngology Department - Otorhinolaryngology Center - São Paulo / Hospital Professor Edmundo Vasconcelos. Centro de Otorrinolaringologia de São Paulo - Hospital Professor Edmundo Vasconcelos

**Keywords:** history of medicine, otorhinolaryngology

## Abstract

Ears, nose and throat have intrigued humanity since immemorial times. Treatments for the larynx, the nose and the ear and also surgeries were practiced by Greek, Hindu and Byzantine doctors. In the 20th century clinical and surgical innovations were incorporated, thanks to new anesthesia techniques, antibiotics, radiology and new technologies. **Aim and method:** show the evolution of this science throughout the times, recognizing important persons in otology, rhinology and laryngology. **Results and conclusion:** Understanding the evolutions in clinical and surgical anatomy, physiology, treatment modalities, and the personalities that lead to these advances is of great importance for the evolution of medical science. Otorhinolaryngology has a very rich history, with important collaborators and personalities in the history of medicine. The specialty was one of the first to use local anesthesia for procedures, pioneer in treatments with devices that recouped hearing and the use of microscopes in surgeries. Few medical specialties had suffered as many changes and scientific developments in these last decades as Otorhinolaryngology had, with the advantage of incorporating technologies such as endoscopy, radiology, microsurgery and information technology.

## INTRODUCTION

Nose, throat and ears have intrigued human kind since immemorial times. Greek, Hindu and Byzantine physicians already practiced treatment and surgeries on the nose, throat and ears. In the XX century, clinical and surgical innovations were incorporated thanks to new anesthetic techniques, antibiotics and radiology being added to the technology.

## OBJECTIVE AND METHODS

To show the development of this science along time, acknowledging important personalities in otology, rhinology and laryngology by means of a literature review.

## OTOLOGY

### Earlier times

In one of the best-known scientific documents, Ebers’s scrolls, from Egypt, there are descriptions of battle wounds on temporal bones, and how they affected hearing and speech. In the Egyptian pharmacopedia, from approximately 1,500 B.C., there is a chapter called: “Medications for the hard of hearing ear “, where one may find treatment for tinnitus, dizziness and hypocusia^1^.

In Greece, physicians and philosophers concocted primitive anatomical studies and theories, in an attempt to explain diseases and how our bodies worked. Alcmaeon of Croton, a physician considered the father of neuroanatomy, imagined that hearing happened thanks to air movements that penetrated the ear and hit the brain in a specific site which was responsible for hearing. He thought that hearing was the result of a concussion that altered the position of the brain, making these airways hit another region^1^.

Empedocles, Greek philosopher, known for numbering the four basic elements (fire, air, earth and water), was the first to describe the cochlea. He called the structure “κoχλoς”, the name of a seashell found in the Mediterranean region. However, his discovery intrigued him more for the perfect and singular shape of that anatomical structure than its function or relation with hearing^1^.

With merely empirical treatments, Hippocrates, was also interested in otology, however himself and his disciples were more concerned with the relations ear infections had with other organs, especially the brain and tonsils^1^.

Aristotle, one of the best-known Greek philosophers, even without anatomical knowledge, created a theory on hearing. He believed there was a resonating space within the inner ear, which vibrated in response to sound. Pure air was implanted in the ear as the person was born, and congenitally deaf people did not have this air implanted there. As time passes, people would lose this pure air, thus reducing hearing^1^.

During the Roman Empire, medicine used Greek knowledge and many of their teachings, and incorporated new discoveries. Cornelius Celsus, in the 1st century A.D., was the first to describe a tonsillectomy, doing it with his own fingers, and some new treatment options for tinnitus, foreign bodies in the outer ear canal and surgeries for external ear canal atresias^1^.

Galeno, personal physician of the Roman Emperor Marcus Aurelius, dissected the ears of dogs and monkeys. Even without a microscope, he was able to dissect the inner ear and called the structure he found “Creta Labyrinth”, admitting his ignorance as to the functioning of such organ^1^.

After the fall of the Roman Empire, very little was added to the existing medical knowledge on otology in the Middle Ages. It was only in the XVI century, during the renascence, that paintings and sculptures helped in anatomical studies, from people like Leonardo da Vinci, Michelangelo, Eustachio, Fallopius, amongst others^1^.

### XVI Century

During this period, Berengario de Capri and Ingrassia from Naples - Italy, described the malleus, the incus and the stapes. Eustachio accurately described for the first time the tensor tympani muscle, identified the corda tympani as a nerve and not as a blood vessel, and the structure that has his name, the tube, described in the paper “De Auditus Organis”, where he even divides it in bony and cartilaginous parts^1^.

Versalio, in 1543, described the oval and round windows, and also the malleus and the incus. Fallopius, from the famous medical school of Padua, discovered and explored the facial nerve canal. It was he who described and named the tympanum, for its similarity with a drum. In the paper “De morbo gallico” he described the high intensity tinnitus that may happen in advanced syphilis stages^1^.

Fabrizi, the most known of Fallopius’s students, published the hearing theory, which merged Aristotle’s idea with a new concept of auditory nerve stimulation. Fabrizi also described for the first time, proper methods for otological surgery lighting, with devices used to guide the sun light or that from candles^1^.

### XVII Century

Anatomical studies of the ear continued during the XVII century. Riolanos, in 1649, described the technique used to do a simple mastoidectomy. The French architect and physician Perrault, in his paper “Debruit” developed the theory that the cochlea was the true hearing organ. He described that the cochlear membrane vibrated under high noises, and it could degenerate as the person aged ^1^.

Under the ruling of Luis XIV, Du Verney, personal physician of the French royal family, published the elegant “Traité de lorgane de loutè” with impressive drawings of the inner ear anatomical structures and also stated that sound was not transmitted by air in direct contact with the round window, but rather by the ossicular chain, directly on the oval window. He proposed a theory of cochlear resonance, even before Helmholtz did. Unfortunately, his theory was based on the inverse pattern that we use today. Comparing the thickness of the spiral sheet with the thinning from base to apex, he proposed that the base would receive low frequency sounds and the apex the high frequency sounds. Du Verney also described the cholesteatomatous otitis media^1^.

### XVIII Century

Right at the beginning of the XVIII century, the French Jean Louis Petit, even before the discovery of the Koch’s bacillus, made the first description of tuberculosis mastoiditis and carried out successfully, mastoidectomies in the treatment of “pus collections in the cancellous tissue of the mastoid apex”. However, Italian physicians, especially those from Bologna medical school, headed by Malpighi1, dominated the otology study at this time.

Valsalva, one of Malpighi’s student, described the “Tractatus de aure humana”, published after his death by Morgani. In 16 years of work at the University of Bologna, Valsalva dissected more than one thousand human heads, and described the ossicular chain discontinuity as a cause of hearing loss. Morgani, who is best known for his anatomical discoveries in the gastrointestinal tract, was also interested in otology. Of the twenty anatomical epistles he wrote, seven were related to the ear. One of them described the auditory effects of minimal anatomical perforations in dogs. In this paper “De causis et sedibus morburum” he proposed the relationship between otitis media and brain abscess^1^.

Cotugno, from Naples, described the perilymph; and Scarpa, from the University of Modena, described the endolymph. At only 24 years of age and still as a medical student, Cotugno published the paper “De acquaeductibus auris humanae”, an impressive study on the cochlear structures and their roles in human hearing. He also identified and located several branches of the auditory nerve and their terminals in the labyrinth, though he never suspected they could be related to a sense other than hearing^1^.

### XIX Century

It was a time of major progress in otology. In the beginning of this century, the French physician Breschet, with the paper “Recherches anatomiques et physiologiques sur lorgane de louïe” brought about some order to the anatomical nomenclature. Breschet demanded some accuracy in the definitions and changed some names and terms, but he accepted the established nomenclature. It was Astley Cooper, in 1801, who carried out the first myringotomy, in an attempt to cure a case of hearing loss caused by Eustachian tube occlusion^1^.

In 1829, Cruveilhier, described a pearl-like tumor in the central nervous system, which was probably a cholesteatoma of the petrous apex and, in 1838, the French Toulemouche described malignant external otitis for the first time^1^.

In Germany, Johannes Müller started pioneering work in experimental auditory physiology. He was the one who coined the term “cholesteatoma” in reference to the presence of cholesterin in the steatoma and its marked destructive power. With his studies, he attracted many students, Helmholtz among them, a physician who became interested in hearing and vision physiology, who, in 1868 described middle ear physiology, based on previous studies by Du Verney^1^.

Dienffenbach, in 1845, reported on the attempt to correct bulged ears, by closing the angle between the concha and the mastoid, by an elliptical resection of skin on the retroauricular region^1^.

Huschke described in the brain stem, the foramen that took his name, when he dissected bird ears. He believed he had found the true end of the auditory nerve fibers; however, Corti made such discovery^1^.

In the middle of the century, Alfonso Corti was invited by anatomist Albert Kölliker to work in the city of Würzburg, where he met Virchow - pathology professor, who introduced Corti to Schroeder van der Kolk and Pieter Harting - Dutch professors. The Dutch used innovative methods with the microscope for anatomical dissections. Corti learned these techniques and used them to study more than 200 cochleas from cats, dogs, pigs, sheep, rabbits, rats and humans, and afterwards he published the paper: “Recherches sur lorgane de loute” in which the Italian physician described, in impressive details, the organ of Corti, located inside the cochlea, the true hearing site^1^, ^2^.

Evenberg, in 1860, reported the first case of sudden hearing loss secondary to mumps and, in 1861, Prosper Ménière described the classic triad of the disorder that has his name: periodic vertigo, hearing loss and tinnitus. Physicians at the time related this disorder to brain congestion, and treated the patients with violent exsanguinations and strong intestinal cleansing medication. Ménière described some cases and treatments of his own personal experience, and showed this disease as an inner ear disorder, and not a brain-related illness^1^, ^3^.

Prosper Ménière, French physician, botanist and historian, interested in otology, published the paper: “Traité des maladies de lourelle” in 1848. Ménière also wrote papers on hearing loss and muteness, and developed ear evaluation methods. He was totally against some barbaric methods practiced by otologists that promised to cure deafness. Ménière thought deafness was incurable and that the patients should be reeducated to learn to live with such challenge. He wrote two texts on this subject: “De la guérison de la surdi-mutité, et de leducation dês sourds-muets”, in 1853 and “Du marriage entre parents, consideré comme cause de la surdi-mutité”, in 1856^3^.

In 1861, Politzer became a professor of otology at the University of Vienna. He performed important work in the areas of otology, compiling and improving theories. In 1865, Politzer published his atlas of otoscopy, expanded and re-published in 1896, besides many other important publications. Politzer started his physiological research in the known laboratory of Carl Ludwig, in Vienna, studying the innervation of middle ear muscles and the opening of the Eustachian tube. He did studies together with Albert Kolliker and Heinrich Müller, in Würzburg in order to learn more about the Eustachian Tube’s function and its functional innervation, and ear histopathology studies with Kolliker^1^, ^4^.

In France, Politzer met with Prosper Ménière and carried out studies with Rudolph Konig and Claude Bernard who investigated the ossicular chain mobility after sound stimulus. He also had the opportunity of studying the famous temporal bone collection of Toynbee, in England^1^, ^3^.

Politzer described important otology treatments, such as the Politzer maneuver or the “Politzerization”, a technique that allowed for better permeability and pressure balance between the middle ear and the nasopharynx^4^.

In 1867, Politzer described myringotomy as ancillary treatment for secretory otitis media. In 1869, Hinton broadened Politzer’s studies on the myringotomy to treat secretory otitis media and, between 1873 and 1885, Hermann Schwartze from Halle published papers systematizing the mastoidectomy technique. He also established simple mastoidectomies indications and techniques^4^.

The Englishman Joseph Toynbee, in 1860, published his paper “Diseases of the ear”, in which he described his collection of temporal bones. In 1874, he noticed that, because of ossicular chain immobilization, the stapes footplate anchylosis was the main cause of deafness in the cases he studied^4^.

In 1875, Mach performed pioneering studies on the rotatory stimulation of human beings, in an attempt to determine the vestibular excitability threshold; and in 1878, Kessel performed the first surgery of stapes mobilization in a patient with stapes footplate anchylosis, a method that had been previously condemned by Politzer and Sibenmann, which fell in discredit until 1953, when Rosen brought it back^4^.

In 1881, Von Tröltsch modified Schwartze’s mastoidectomy and called “sclerosis” the stapes footplate anchylosis described by Toynbee. In this same year, Retzius carried out a pioneering work on the technique of labyrinth micro-dissection. In 1883, Politzer described auditory dystrophies by scarring processes and, in 1887, he changed the term Von Tröltsch had used: “sclerosis” to otosclerosis, because it was an ear anchylosis of bony nature. Afterwards, Ostmann proposed a new term: “otospongiosis”^4^.

In 1889, Emanuel Zaufal and Stacker described the radical mastoidectomy, with the removal of the osteitic process. However, their surgeries made all patients deaf. Kuster defined the indications for mastoidectomy and advocated the removal of the postero-superior wall of the external acoustic meatus in order to broaden the surgical field ^4^.

In 1892, Ewald established the labyrinthine origin of nystagmus and, in the following year, in Brazil, Guedes de Mello performed in Rio de Janeiro the first antrostomy, technique described by Wilde that curetted and removed bone sequesterings. In 1894, Politzer described the hypoacusis without a known cause, stressing the possibility of external factors involvement, such as cold, local trauma or barotrauma and, in 1896, Alderstron made a fenestration, perforating the stapes footplate by using a burr driven by a dentist’s motor^4^.

In the following year, Passow electronically perforated the promontory by the retroauricular approach, and João Marinho published the thesis called “Da trepanação da apófise da mastóide nos casos de otite média” (Mastoid apophysis trepanation in cases of otitis media), based on the procedures carried out by Henrique Guedes de Mello^4^.

### XX Century

Otology and laryngology merged in order to form Otolaryngology. A time of major progresses in this field. Alt, from Vienna, built the first electrical hearing aid, called microtelephone, built from an amplifier and a coal microphone, a large size device that bothered the users. Politzer, already retired from medical practice, wrote and brought back fantastic works on the history of otology^5^.

In 1901, Perry, Scottish physician, opened the inner ear canal of a patient with Ménière’s disease, cut the nerve and started the VIII cranial nerve neurotomy. In the post operative, the patients had total facial paralysis, and despite such major complication, Lannois and Jaboulay carried out this procedure in France in the same year^4^.

In 1910, Bondy described a surgical technique for apical cholesteatoma with ossicular chain preservation and perforation of the tympanic pars flacida. In this same year, Robert Bárány successfully trephinated the posterior semicircular canal without opening the antrum. In 1911, in Bahia Medical School, the first chair of Otorhinolaryngology was created, and Eduardo Rodrigues de Moraes was the first holder of such chair in Brazil. In 1912, Kisch described a tympanoplasty for the first time, in a paper published in the “Proceedings of Royal Society”. In 1913, Jenkins trephinated the horizontal semi-circular canal, in an attempt dry the labyrinth and, in 1914, Bárány received the Nobel Prize because of his work on vestibular apparatus physiology and pathology. In 1918, Diniz Borges published a pioneering thesis in Brazil on topics related to the vestibule and, in 1919; Marcel Lermoyez described the syndrome that carried his name with the symptoms: slow and progressive tinnitus and hearing onset, usually unilateral, followed by nausea and vomit^4^.

In1920, Gillies was the first to use cartilage in remodeling the framework in cases of pinna reconstruction. In 1921, the Swedish Carl Nylen introduced the monocular microscope for ear surgeries. In the same year, Lermoyez, Boulay and Hautant published studies on chronic otitis media. In 1922, Fletcher and Wegel introduced the audiometric exam in screening patients for hearing loss, Schutt and Meyers, using Du Bois-Raymond’s experiment, were the first to analyze nystagmus. In this same year, the Brazilian Mário de Ottoni Rezende published an important monograph on neurophysiology and pathology of the vestibular apparatus. In 1926, the French Georges Portmann performed the Ménière’s disease surgery, later on perfected and disclosed by the North American William House. In the following year, Cecil Alport described the history of a family with deafness and kidney disease, currently known as Alport’s syndrome. In 1929, Lüscher described the middle ear acoustic muscle activity, directly observing a patient with tympanic membrane perforation^4^.

In 1932, Balance and Duel introduced the facial nerve decompression technique by opening its temporal bone canal. In 1933, Mário Ottoni de Rezende and Homero Cordeiro started editing the Journal Otolaryngológica de São Paulo, which later became the Revista Brasileira de Otorrinolaringologia (Brazilian Journal of Otorhinolaryngology). In 1934, Schuster measured for the first time the middle ear impedances and, in 1938, in an alternative technique, under local anesthesia, Julius Lempert, made a small fenestration on the lateral semicircular canal in order to guide the sound straight to the inner ear, isolating the otosclerosis site. In the same year, Jefferson and Smaley described a congenital cholesteatoma in the petrous portion of the temporal bone^4^.

In 1940, Boettcher introduced the electrical burr for mastoid surgery. Such technique was much rejected by some otorhinolaryngologists when it was first introduced, because they believed that such high burr rotations could cause acoustic trauma. In the following year, the jugular glomus was first described by Stancey Guild, which was classified in 1945, by Rosenwasser and Sadao Otani^4^.

In 1953, Zeiss Optical Company, in the USA, introduced modern microscopic ear surgery with the development of the binocular microscope. Fritz Zöllner and Horst Wüllstein reported many cases of successful treatments of tympanic perforations and middle ear chronic suppurative processes with the use of binocular microscopes. Rosen reintroduced, in the annual meeting of the American Triological Society, the technique of stapes mobilization for the treatment of otosclerosis and William House described the first case of cholesteatoma in the middle ear cleft, seen through the intact tympanic membrane. In the following year, 1954, Armstrong introduced a polyethylene tube in the tympanic membrane, in order to treat cases of serous otitis media; and Clerc and Batisse systematized the temporal bone surgical approach through the middle fossa, enhanced and popularized by William House in 1961^4^.

Otorhinolaryngologists started to have better control and knowledge about the use of the microscope and the internal acoustic meatus area. This site became a common point between otolaryngologists and neurosurgeons, and the pioneering work of William House, in Los Angeles, contributed for the successful removal of pontine-cerebelar tumors with the use of microscopes^4^, ^5^, ^6^.

Despite all the scientific knowledge developed in this area, the natural degenerative process of human life remains. Tinnitus, vertigo and hypoacusis remain as important causes of quality of life reduction, especially in the elderly. Such fact and the desire to help children, who are already born with hearing problems, pushed the development of auditory prosthesis, cochlear and brainstem implants^4^, ^6^.

In 1967, Sohmer and Feinmesse, attempted to record the electrical activity of the human brainstem, evoked by acoustic stimulus, which became known as early potentials of the auditory pathways. In 1968, Aran and Lê Bel, in Bordeaux, France, established the basis for electrocochleography as an objective method for auditory evaluation. In 1970, Jewet, Romano and Wilinston proved the origin of brainstem potentials, standardizing the recording technique and the nomenclature still used today for the waves. In 1972, Jean Marie Aran published electrical response observations that can be seen between the cochlear bony promontory and the ear lobe by means of electrocochleography, introduced in Brazil in the following year by Yotaka Fukuda and Ossamu Butugan^4^.

The concept of cochlear electrical stimulation producing hearing is nothing new. The Italian Alessandro Volta, known for having invented the electrical battery, had already performed experiments placing metal plates in his ears and connecting them to electricity in 1800. The experience was not pleasant, but he reports he heard the noise of boiling water before passing out. A Russian group carried out a true stimulation of the auditory nerve for the first time in 1934, and the modern progress of cochlear implants started back in 1960 by groups of otorhinolaryngologists and engineers in France, Germany, Austria and the United States^6^.

In Brazil, Pedro Luiz Mangabeira Albernaz carried out the first cochlear implant, in 1977. Now, brainstem implants, in which persons with auditory nerve impairments can recover part of their hearing, were also incorporated to otorhinolaryngology by William House, in the United States and, in Brazil, by Ricardo Ferreira Bento^4^.

The future of research in otology is coming at a furious pace, especially in the field of molecular genetics. In the year of 2005, many deafness-related genes were identified by the Genome project and genetic engineering technology with DNA alterations, besides stem cells in the repair and regeneration of hair cells are already being investigated^6^.

## LARINGOLOGY

### Early years

Larynx and pharynx surgeries and treatment reports date back from Egyptian, Hindu and Greek physicians. The oldest reference in laryngology is a drawing found in medical tombs in the planes of Saqqara, Egypt, from approximately 3,600 years B.C. The image seems to portray a tracheostomy. In India, medical documents called “Sushtrata”, from 300 B.C. and “Charaka”, from the year 100 B.C. had chapters describing drugs and treatments for voice disorders, suggesting some anatomical knowledge of the throat and larynx as the origin of our voice^1^, ^5^, ^6^, ^7^.

Aristotle was the first to mention the larynx in his book: “Historia Animalium”, Book I, chapter XII of the year 350 B.C. in which he described: “the neck is the part between the face and the chest. Anteriorly is the larynx. Speech and breathing happens through it, which is protected by a structure known as the windmill.” Erasistratos, in the year 290 B.C. described the function of laryngeal muscles and Galeno, in Rome, in the II century A.D. in his treaty “De usu partium corporis humini” already discussed laryngeal functions^5^.

One of the first written reports on larynx treatment and surgeries date back from Macedonia. Historians report a tracheostomy made by Alexander the Great himself, who saved the life of an agonizing soldier by sticking the tip of his spear in the region described by Aristotle as “the windmill”, probably the cricoid cartilage^5^.

### XVI, XVII and XVIII centuries

Artists such as Leonardo da Vinci and Michelangelo performed some dissections in human cadavers and reported detailed descriptions of laryngeal function. The first laryngectomy, a precursor of modern tracheostomy, seems to have been performed by Musa Brasavola in Italy, in 1545. Giovanni Morgani in the paper “Adversaria Anatomica Prima” brought about detailed illustrations of the larynx. Ferrein, in 1741 was the first to publish the term “vocal cords”. He compared the structures to the cords of a violin, activated by the contact with an air column. Bertin, in 1745, brought this new concept that the structures described by Ferrein were, in fact, folds, and not cords^5^.

### XIX century

The barrier preventing laryngology to develop was the incapacity to directly examine the larynx. Clinical laryngology made it possible, by means of a number of favorable developments. Lighting methods and observation through mirrors, local anesthesia, aseptic surgery practices and an increase in our knowledge regarding cellular pathology made possible this new medical practice^5^, ^6^, ^7^.

In 1806, Bozzini developed an angled speculum with a mirror, used to examine the most varied human cavities^5^.

In 1837, physiologist Johannes Muller, in Berlin, expanded Ferrein’s results, as he analyzed the movement of vocal cords in cadavers^5^, ^6^.

The English physician Benjamin Ebbington carried out a laryngoscopy with a device called “glottiscope” in 1829, however, the first successful use of the mirror to inspect the larynx was not made by a physician, but rather by a Spanish Music Professor - Manuel Garcia in 1854. With a small mirror, used by dentists and proper lighting, he could see the functioning of his own vocal cords with breathing and vocalization. He published numerous books about voice and developed his own laryngoscopic technique^5^.

Before using the small mirror, laryngoscopy attempts were made with a device called Avery’s laryngoscope, but unsuccessfully. Mackenzie, in 1865, wrote about these frustrated attempts on the use of Avery’s laryngoscopy in the paper “The use of the laryngoscope in diseases of the throat”^5^.

After descriptions by Garcia, Carl Ludwig Türck developed laryngeal mirrors and used them not only to examine his own larynx, but also to observe laryngeal pathologies in his patients using sunlight, only during spring and summer in Europe. Türck developed his studies with Ernst Brucke, who worked with Johann Nepomuk Czermak, from Budapest. Czermak was very interested in this technique and adapted artificial lighting for laryngeal studies during fall and winter, what made him publish numerous uses for the mirror with lighting in the exam of patients with laryngeal pathologies, but without mentioning in his work the collaboration from Türck^5^.

The two doctors became enemies, and Türck even questioned the work of Czermak. The rivalry between these two researchers allowed for a fast progress in this field, and it also made Czermak take the initial steps in rhinology. A student of Czermak’s, Friedrich Semeleder, developed rhinoscopy together with his professor. Now, Türck had, among his pupils, Schnitzler who, in 1895 created an impressive atlas of laryngology, used until current times in the University of Vienna^5^.

In England, Sir Morrell Mackenzie was fascinated with the descriptions made by Czermak. He redesigned the laryngeal mirror and baptized it “laryngoscope”. He also designed instruments for laryngeal biopsy, by using indirect laryngoscopy. Gustav Killian, in Berlin, also developed a device for laryngoscopy and a device for laryngoscope holding. Lynch modified this device and made it popular in the United States. Near the end of the century, Chevalier Jackson, in Philadelphia, developed a distal lighting method for the endoscopy equipment. Jackson, with great enthusiasm, dexterity and communicative skills was one of the person’s responsible for the expansion of endoscopic applications in the diagnosis and treatment of laryngeal lesions^5^, ^6^.

Even with the works of British otologist James Yearsley “Deafness cured by cleaning out the passages from the throat to the ear”, of 1839 and the treaty “On throat deafness and the pathological connections of the throat, nose and ear”, of 1853, the specialties of otology and laryngology existed separately: otology practiced by surgeons and laryngology by clinicians, who prescribed treatments for acute laryngitis with the inhaling of benzoin vapors and tolu balsam, and also formulations used to prepare singers before concerts, which could also be used in cases of unilateral vocal fold paralysis, which was coca leaf tea^5^, ^6^.

However, laryngologists, performed small surgical procedures, such as the removal of minimum lesions in the larynx and pharynx using curved mirrors and forceps^5^, ^6^.

Local anesthesia with cocaine in laryngeal surgeries started to be used by ophthalmologist Karl Koller and laryngologist Edmund Jelinek, in Vienna, who removed a laryngeal polyp from a patient and improved the pain of another patient with tuberculosis in his larynx^5^, ^6^.

In 1862, the German Von Bruns reported the successful removal of laryngeal polyps; however, one of the major problems for performing small laryngeal surgeries was access and the surgical drape, a problem that was solved by different methods along time. In 1879, Reichert described an epiglottis retractor^5^.

Other larger lesions in these regions needed to be treated by surgeons, and Sir. Felix Semon was the first laryngologist-surgeon of London. At this time, he already did laryngeal-fissure surgeries with his student Sir St. Clair Thomson, for laryngeal cancer in initial stages. Thomson was the first to correlate these lesions with smoking and followed patients, recording two intraoperative deaths and a survival rate of 76% in 3 years, in a series of 74 patients in 1906^5^.

The first total laryngectomy was carried out in 1873, in Vienna, by surgeon Theodor Billroth. The 35-year-old patient survived the procedure and lived for seven more months. The major complication described by Billroth in this patient was aspiration and difficulties swallowing. Gluck, Billroth’s student, solved this problem by doing a surgical technique in which he separated the larynx from the trachea, opening the neck’s skin and suturing the trachea’s orifice directly on this opening. Gluck also had knowledge on the removal of lymph node cells with metastatic involvement during the surgery to remove the primary tumor. By doing so, he obtained the best results^5^, ^6^.

Carl Gussenbauer developed a vocal prosthesis in 1874 to be used by patients submitted to Billroth’s laryngectomy. In 1900, Nicholas Taptas, a Turkish doctor, rehabilitated a laryngectomized patient using a connection between the trachea and a pharyngeal fistula created, making the patient speak as she occluded the tracheal hole in her neck^5^.

One of the most curious facts in the history of laryngectomies was the sickness of Prince Frederich, from Germany. In January of 1887, the Prince started to have dysphonia, initially attributed to a cold. Inhalations and gargling were not efficient, and his physician, Wegner, called Gerhardt, the famous laryngologist from Berlin^8^, ^9^.

As he saw a hyperemic node in the left vocal fold, during laryngoscopy, Gerhardt tried to remove it with a metal loop, but he was unsuccessful. Another frustrated attempt happened with the scalpel. He finally managed to cauterize it with the electrical cautery; however, the wound increased in size, even after weekly cauterizations. The vocal cords were mobile, which at the time ruled out the possibility of malignancy. The prince was advised to spend 15 days resting in the mountains, however his dysphonia worsen. In May, Gerhardt decided to call a surgeon, Von Bergmann, to “cut it out”. When Queen Victory from England, received a letter from her daughter, princess Viki, Frederich’s wife, about his disease, she consulted with her private physician, James Reid, who immediately indicated the most renowned otorhinolaryngologist of England, Morrell Mackenzie, for the procedure. Mackenzie went to Germany right away. He was the most famous otolaryngologist from London, however, his throat hospital, still in Goldn Square today, was not well regarded by the Royal College of Surgeons of England^8^, ^9^.

Amidst all this commotion, dozens of physicians present, Mackenzie operated the prince, under anesthesia by chlorophorm, on May 21 of 1887, removing part of the tumor through laryngoscopy. The surgical specimen was sent to Virchow, who examined it and declared it was not a tumor^5^, ^6^, ^7^, ^8^, ^9^.

Then, they called a round table to discuss the case, with Mackenzie, Von Schrötter from Vienna, Krause from Berlin and Moritz Schmidt from Frankfurt. They came up with two solutions: total laryngectomy, which at the time was almost murder, or a palliative traqueostomy, to improve his breathing. The Prince chose the second alternative^5^, ^6^, ^7^, ^8^, ^9^.

Bramann, specialist in tracheotomies in children with diphtheria, performed tracheostomy in January of 1888. On March 09, 1888, Frederich’s father, Emperor William I, died and, the aphonic new Emperor Frederich III took the train to Berlin to take over the imperial throne^8^, ^9^.

His clinical condition worsened; and Mackenzie exchanged the tracheostomy cannula frequently and with increasing difficulty, until one day, during a cough spell, the Emperor coughed his own trachea by the orifice, and died 93 days after having taken the throne^5^, ^6^, ^7^, ^8^, ^9^.

### XX Century

Greenfield Sluder, in the United States, popularized the use of the “guillotine” to perform tonsillectomies; Brunings, in Germany and Jackson, in the United States, started to use the monocular microscopes to perform larynx surgeries during the 1950’s. With the invention and widespread use of binocular microscopes, new surgical techniques for laryngeal procedures were introduced with the use of Yankauer’s laryngoscopes, which used binocular magnifications and which were redesigned by Jako in 1970^5^.

Nuclear technology also brought about progress in the medical field, especially with radiotherapy for the treatment of malignant lesions in the larynx. Problems such as mucositis, skin burns, dosage and scope of action were being solved by piecemeal^5^.

Optic fibers endoscopes were developed in 1954, by Hopkins, and brought about a new era in endoscopy with flexible fibroscopes, used to examine the larynx, nasopharynx, nose and pharynx^5^, ^6^.

Currently, larynx examination has been greatly developed thanks to the pioneer work of physicians such as Karl Storz and Hopkins, together with new techniques being developed for treatment and surgery^5^, ^6^.

In the last 30 years, laryngology has gradually evolved with the collaboration of head and neck surgeons, radiotherapists, oncologists and other specialists^6^.

Thyroplasties, oral cavity reconstructions, flaps and microvascular anastomosis are carried out with the support of new technology.

The concept of phonosurgery, as a procedure to improve or reestablish voice, introduced by Von Leden was restricted to laryngeal microsurgery to remove lesions on the top of the vocal folds, which projected to the glottal space. Minimum structural alterations were not part of the diagnostic routine. Currently, with more sophisticated microsurgery material and with better physiopathological material, endolaryngeal microsurgery has become more efficient. Surgeries of the laryngeal framework, thyroplasties, published by Isshiki since 1976, were popularized at the end of the 80’s, thanks to courses given at the North American Academy^6^.

The growing sophistication and evolution of endoscopes, with modern optic fibers, combined with more sensitive stroboscopes, have led to a greater understanding of how the vocal cords work and a new era in laryngology: voice quality analysis. Working closely with singers and people who use their voices professionally, these tools help diagnose early on small lesions and perform microsurgeries of the larynx, phonosurgeries, which allow the removal of these lesions in just a few minutes.

In 1998, the first larynx transplant was carried out in Cleveland; however, there were numerous problems, especially related with organ rejection by the patient’s immune system^6^.

## RHINOLOGY

### Early Times

Nasal region studies, the olfactory function and the knowledge about the paranasal sinuses date back from the most remote times, as well as the attempts to treat disorders of this area.

Egyptian physicians were the precursors of nasal surgeries. They used instruments to remove the brain through the nose, as part of the mummification process^10^, ^11^.

The first report in the world medical literature of a nasal exam dates back to the sixth century before Christ, in the Hindu document “Suchruta-samhita” in which they describe a tubular nasal speculum, made of Bamboo tree, used for tonsillectomies and surgeries to remove nasal polyps^10^.

Hippocrates, in the V century B.C., already described therapeutic methods for nasal lesions. He classified them from simple bruises in soft tissues all the way to complicated fractures, indicating detailed treatment for each case, from the use of bandages and braces made of olive tree branches all the way to nasal bone and cartilage reconstructions. Hippocrate’s texts reflected the interest in nasal injuries, which were common accidents at the time, both in soldiers in battles as well as in athletes in competitions in the ancient Greece. These treatment modalities were adapted and they influenced the medical practice until the middle ages^10^, ^11^.

### XV Century

Although Hippocrates had already identified parts of the nasal anatomy, it was only in the XV century that the nasal structures were described.

Leonardo da Vinci drew the nasal conchae and the paranasal sinuses, in 1489. However, these drawings were only found in 1901, in Milan. George Thomas first described the posterior insertions of the middle conchae in the paper “Anatomiae pars prior”, in 1536^10^, ^11^, ^12^.

The first book entirely dedicated to describing the surgical techniques for rhinoplasty was published in 1597 with the title “Treaty on Rhinoplasty”. The author was Gaspare Tagliacozzi, Professor at the University of Bologna, who was very experienced in this topic; he proposed new techniques to rotate flaps over the nasal pyramid^10^, ^11^, ^12^.

In 1651, Highmore, in England, described the maxillary sinus, and for a long time such structure was known as the Highmore’s antrum^12^.

In addition, in the Middle Ages, obscure functions were attributed to the paranasal sinuses, such as to store oils to lubricate the eye movements, or a drainage space for malignant spirits in the brain. These functions brought names to the paranasal sinuses, such as “la cloaca del cerebro”, according to the Spanish physician Sansovino, in the XVI century^12^.

In 1660, Schneider, in the city of Wittenberg - Germany, was one of the first to imagine that the mucus present in the paranasal sinuses was not a product of the brain, but rather it came from the very paranasal structures^10^, ^11^, ^12^.

### XVII and XVIII Centuries

During the XVII and XVIII centuries, the major scientific discussion about the nasal region regarded the function and objective of paranasal sinuses. Many diseases were attributed to such structures, from halitosis to facial acne, of which advocated treatment was total or partial middle turbinectomy.

In England - 1707, Drake and Cowper, reported some cases of halitosis caused by maxillary sinus suppuration. They treated the patient by pulling out teeth, thus opening the maxillary sinus through the alveolus^12^.

In France - 1765, Jourdain, tried to cure maxillary sinuses suppurations by irrigating the structures through their natural ostium, present in the middle meatus, but he was not very successful. In 1743, Lamorier was already opening the maxillary sinus through the oral cavity; however, he only published descriptions of his work in 1768. Lamorier’s method of opening the maxillary sinus through the tooth socket remained the standard procedure for a long time^12^, ^13^.

### XIX Century

In Berlin - 1841, Henle did microscopic studies and differentiated various epithelia, especially describing the function of the ciliated epithelium found lining the nose and the respiratory tract^12^.

In Vienna - 1886, Mikulicz-Radecki, was the first to describe the opening of the maxillary sinus through the inferior meatus. In the USA - 1893, Caldwell published his method, which consisted in opening the sinus through the canine fossa, removing the mucosal membrane and opening the lateral wall of the inferior meatus. In Berlin - 1896, Boenninghaus was one of the first European physicians to follow Caldwell’s technique; however, he modified it by placing a mucosal flap covering the opening. Without being aware of Caldwell’s works, Luc, in Paris - 1897, reported his own method, which was practically identical to that of his American counterpart^12^.

However, current knowledge regarding the anatomy of these structures is greatly due to the works of Emil Zuckerkandl, from Austria, who in 1870 described details of the nose and paranasal sinuses in anatomical studies, thus opening a new field for scientific and surgical knowledge in this area. The decades that preceded the XX century boosted studies of surgical and sectional anatomy, with scholars such as Grunwald, Onodi, Hajek, witnessing the birth of rhinology as a specialty, making up the basis of our current concepts of diagnosis and treatment of the nasal cavities’ and paranasal sinuses’ diseases^12^.

Rhinology also enjoyed a major thrust forward, especially in the areas of diagnosis and surgery when endoscopy was created, credited to Philipp Bozzini in 1806. Czermak, who for the first time mentioned the term “rhinoscopy”, popularized the use of a nasal speculum and, in 1879 the use of an endoscope in rhinoscopy^10^, ^11^, ^12^, ^13^.

### XX Century

Contrary to expectations, the century started with a stalling in the area of rhinology, such fact was due to antibiotics, which greatly reduced the need for surgery in the paranasal sinuses. Moreover, laryngology and otology developed greatly and reduced the interest in rhinology, which was restricted to the correction of nasal septum deviations, fractures, removal of nasal polyps and maxillary sinus flushing through the canine fossa^11^, ^12^.

Nonetheless, some physicians remained interested in this area and developed surgical techniques to approach and treat lesions in the nose and paranasal cavities.

In 1901, Hirschmann used a modified endoscope to inspect the maxillary sinus. The Brazilian Ermiro Estevam de Lima became internationally known for developing a transmaxillary approach to the ethmoidal and sphenoidal sinuses, to which he developed the curette that carries his name. This surgical approach soon became worldly known as “Ermiro de Lima” procedure. He was also the founder of the Brazilian Society of Rhinology, in 1974, which had Roberto Machado Neves Pinto and Sérgio de Paula Santos as some of its first members. In 1912^4^, Harvey Cushing started the trans-sphenoidal approach in neurosurgery. Surgeries to access the frontal and ethmoid sinuses were also described by Lynch in New Orleans, in 1921^13^.

In 1926, John Baird, the inventor of television, patented the idea of transmitting images through flexible glass fibers. These ideas influenced Harold Hopkins, who invented scopes in 1948; Basil Hirschowitz, performed the first digestive endoscopy with flexible glass fibers optics and Karl Storz^11^, ^12^, ^13^.

In the middle of the century, the microscope started to be used also in nasal surgeries, which brought about major progress to the surgical technique. A greater knowledge regarding immunology, made physicians differentiate and understand allergic and non-allergic processes. A key for the development of knowledge on the anatomy, physiology and pathology of paranasal sinuses is credited to professor Walter Messerklinger and his successor, professor H. Stammberger, from Austria, in their work about the aeration of the anterior ethmoidal cells, which was key to understanding drainage and aeration of paranasal sinuses, as well as the anatomy of the lateral wall of the nose and its mucociliary clearance^11^, ^12^.

Messerklinger brought back the use of endoscopes, using it for diagnostic and surgical procedures in the nose^11^.

The use of new technologies caused an advance in endoscopic techniques, especially with the development in 1954 of the optic fiber endoscopes by Storz Fiberoptic Company. Such progress, added to CT scan - developed in 1969 by Geoffrey Hounsfield made it possible to have a detailed analysis of the nasal cavity, especially the lateral wall and the ostium-meatal complex^11^, ^12^, ^13^.

Reynolds and Brandow refined surgical treatment of refractory sinusitis with the use of antrostomies carried out under endoscopy. David Kennedy, Heinz Stammberger, Wolfgang Draf and, in Brazil, Aldo Stamm were major participants in the popularization of the use of modern endoscopy in nasosinusal surgeries.

CT scan also helped much in the development of functional endoscopic sinus surgery, realized by Kennedy^11^, ^12^.

Gerard Guiot was the first to use endoscopy for a trans-sphenoidal approach in neurosurgery. This happened in 1970 when, together with Bushe and Halves, they reported the use of endoscopes to access pituitary lesions, which were previously resected under microscopy; however, Jho and Carrau were pioneers in performing purely endoscopic neurosurgeries, where both access and resection were carried out under endoscopy^11^, ^12^.

Another factor that brought about great interest in rhinology, was the unveiling of rhinoplasties, a work carried out by the American Academy of Reconstructive and Facial Plastic Surgery, founded in 1964, and the European Academy of Facial Surgery (Joseph Society), founded in 1977. This brought to otorhinolaryngologists a playing filed that before belonged only to plastic surgeons^6^.

Skull-base surgeries and neurosurgeries with endonasal access, different approaches in the treatment of tumors and diseases located in this region also created, just as it happened in otology, another common ground for otorhinolaryngologists and neurosurgeons^6^.

## CONCLUSION

Historical knowledge about the developments in anatomy, physiology, clinical and surgical treatments; and that of the personalities that led such progresses is of the uttermost importance for medical science to develop increasingly more.

Otorhinolaryngology has a very rich history, with important collaborators and personalities in the history of medicine in general. Our specialty was one of the first to use local anesthesia to perform procedures, a pioneer in using prosthesis to recover hearing and was the first to use microscopes and endoscopes in surgeries.

Few medical specialties suffered so many changes and scientific progress in the last decades as Otorhinolaryngology did, which had the advantage of incorporating technology in endoscopy, radiology, microsurgery and the use of information technology.
